# Aryl Hydrocarbon Receptor and Stem Cells

**DOI:** 10.1155/2017/4602854

**Published:** 2017-01-09

**Authors:** Thomas A. Gasiewicz, Kameshwar P. Singh, Fanny L. Casado

**Affiliations:** ^1^Department of Environmental Medicine, University of Rochester Medical Center, Rochester, NY 14642, USA; ^2^Instituto de Ciencias Omicas y Biotecnología Aplicada, Pontificia Universidad Católica del Perú, Avenida Universitaria 1801, San Miguel, Lima 12, Peru

From the biochemical point of view, the aryl hydrocarbon receptor (AHR) is a highly conserved, developmentally regulated, and ligand-activated member of the bHLH/PAS family of transcription factors. The AHR was originally discovered because of the human and animal toxicity of its most potent and persistent ligand, 2,3,7,8-tetrachlorodibenzo-p-dioxin, usually just referred to as “dioxin.” The canonical understanding of the AHR indicates that it is present in the cytosol as one of the components of a multiprotein complex containing the immunophilin-like protein XAP2/AIP/ARA9, the 23 kDa cochaperone protein p23, and two molecules of HSP90. After ligand binding, it translocates to the nucleus forming a heterodimer with HIF-1beta a.k.a. ARNT and together they can activate gene transcription.

Later studies demonstrated that dioxins also act as endocrine disruptors [1] and as carcinogens with tumor-promoting properties and indirect roles in chemically induced carcinogenesis during initiation, progression, and metastasis. Epidemiological studies linked human exposure to environmental AHR ligands to increased incidence of diverse cancers [2], as well as diabetes and obesity [3]. While more recent research has focused on the physiologically relevant AHR ligands which might be expressed in diseased tissues or active innate and adaptive immune, the physiological relevance of the AHR without ligand binding was not fully appreciated until studies showed a link with stem cells of various tissues of origin [4]. Furthermore, AHR completely left the specialized realm of toxicological research when Boitano et al. [5] showed the pharmacological potential of the receptor by promoting expansion of hematopoietic stem cells in vitro.

In this special issue, original reports as well as thorough reviews are presented addressing the different contexts provided by the tissues and making AHR in stem cells prone to respond to different stimuli such as ligand binding and oxidative stress. Also, it is evident that current biotechnological approaches are providing a more comprehensive understanding of AHR behavior in multiple cell types which is now possible thanks to genome editing tools. With worldwide banning of polyhalogenated pesticides usage and decreases in cigarette-smoke exposures, AHR research leads the efforts to a more sophisticated understanding about the meanings of toxicity in an era of stem cell manipulation.

*Thomas A. Gasiewicz* *Kameshwar P. Singh* *Fanny L. Casado*
